# Trends in Leisure-Time Activity Participation Among Young-Old Adults in China

**DOI:** 10.1093/geroni/igab046.1133

**Published:** 2021-12-17

**Authors:** Wei Zhang, Qiushi Feng, Huashuai Chen

**Affiliations:** 1 University of Hawaii at Manoa, Honolulu, Hawaii, United States; 2 National University of Singapore, Singapore, Singapore; 3 Xiangtan University, Durham, North Carolina, United States

## Abstract

Engagement of leisure activities is highly associated with health and wellbeing in later life. In this study, we examined the trends of leisure activity engagement in young-old adults aged 65–74 in China for a 16-year period. Panel data for a nationally representative sample of young-old adults were obtained from the 2002–2018 Chinese Longitudinal Healthy Longevity Survey. Findings revealed that, compared with 2002, young-old adults in subsequent years were less likely to engage in any form of social leisure activity. The odds of participating in social events, regular exercise, and outdoor activities decreased most prominently over time, while downward trends in tourism and joining outdoor activities showed signs of reversal post-2014. In contrast, trends for engaging in home-bound and solitary leisure activities generally increased. The future elderly in China have generally tended towards solitary leisure activities over time and public health interventions are required to reverse such trends.

